# Genome-Wide Identification and Expression of the *ERF* Gene Family in *Populus trichocarpa* and Their Responses to Nitrogen and Abiotic Stresses

**DOI:** 10.3390/life15040550

**Published:** 2025-03-27

**Authors:** Mingwan Li, Jun Zou, Qian Cheng, Ran Fu, Dangquan Zhang, Yong Lai, Yuanyuan Chen, Chaochen Yang, Wentao Hu, Shen Ding

**Affiliations:** 1 College of Forestry, Henan Agricultural University, Zhengzhou 450046, China; limingwan3@126.com (M.L.); zjunky@163.com (J.Z.); 17622387056@163.com (R.F.); zhangdangquan@163.com (D.Z.); yonglai@henau.edu.cn (Y.L.); cyuan091@163.com (Y.C.); ycc0426@henau.edu.cn (C.Y.); 2 College of Horticulture and Forestry Science, Hubei Engineering Technology Research Center for Forestry Information, Huazhong Agricultural University, Wuhan 430070, China; 17786579574@163.com; 3 State Key Laboratory of Conservation and Utilization of Subtropical Agro-Bioresources, Guangdong Laboratory for Lingnan Modern Agriculture, Guangdong Key Laboratory for Innovative Development and Utilization of Forest Plant Germplasm, College of Forestry and Landscape Architecture, South China Agricultural University, Guangzhou 510642, China; hwt@scau.edu.cn

**Keywords:** *Populus trichocarpa*, ERF family, bioinformatics analysis, gene expression

## Abstract

The ethylene response factor (ERF) family is a prominent plant-specific transcription factor family, which plays a crucial role in modulating plant growth and stress tolerance. In this study, a total of 210 *ERFs* were identified in *Populus trichocarpa*, comprising 29 AP2 (APETALA2) subfamily members, 176 ERF subfamily members, and 5 RAV (related to ABI3/VP1) subfamily members. The duplication events of the *PtERF* family members exclusively occurred within the subfamilies. A total of 168 duplication pairs were found among 161 *PtERF* genes, and all of them were fragment duplications. Gene structure analysis revealed that most ERF subfamily members only had one exon without introns, the AP2 subfamily members had six or more introns and exons, and RAV subfamily members lacked introns except for *PtERF102*. Considerable cis-acting elements associated with plant growth and development, stress response, hormone response, and light response were detected in the promoters of *PtERF* genes. The expression levels of *PtERFs* were highest in roots across tissues and in winter among seasons. Furthermore, the nitrate and urea stimulated the expression of *PtERF* genes. The co-expression network analysis based on *PtERFs* indicated their potential roles in hormone signaling, acyltransferase activity, and response to chemicals. This study provides novel insights into investigating the role of *PtERFs* in environmental stress in poplar species.

## 1. Introduction

Transcription factors (TFs) are proteins located in the cell nucleus that mediate DNA transcription [1]. They can specifically interact with cis-acting elements in promoter regions, thereby regulating the transcriptional expression of downstream genes [2]. Plants growing in natural environments are exposed to various biotic and abiotic stresses, such as fungi, bacteria, viruses, pests, salinity, drought, high temperature, and low temperature [3,4]. These stresses have a detrimental impact on plant growth and productivity. The expression of genes in plants can be induced by various stresses, and the products of gene expression not only contribute to stress tolerance but also regulate gene expression and signal transduction in stress responses [5]. There are approximately 1300–1600 genes encoding TFs in *Arabidopsis* and rice, which act as pivotal regulators involved in plant stress tolerance by modulating the expression of target genes [6].

The ethylene response factor (ERF) family is one of the largest TF families in plants, and it plays crucial roles in plant growth and development and hormone regulation, as well as responses to biotic and abiotic stresses [7,8]. The ERF family is classified into four subfamilies, namely AP2 (APETALA2), ERF, RAV (related to ABI3/VP1), and Soloist, based on the number of AP2 domains and sequence similarity [9]. The AP2 subfamily comprises two AP2 domains and plays a crucial role in regulating plant growth and development [10]. The RAV subfamily is characterized by an AP2 domain and a B3 domain and is involved in the response to hormones such as ethylene and brassinolide [11]. The ERF subfamily contains one AP2 domain and can be further divided into the DREB (Dehydration-Responsive-Element-Binding Factor) and ERF subgroups [12]. Members of the ERF subgroup bind to the ethylene response element (ERE) (GCC-box, core sequence AGCCGCC), while members of DREB subgroups bind to a DRE element (core sequence A/GCCGAC) [13]. The Soloist subfamily contains an AP2 domain, but its gene structure differs significantly from that of other subfamilies [14]. In addition, *ERF* genes in *Arabidopsis* were categorized into 10 branches based on their phylogenetic relationships [6].

The ERF transcription factors play a crucial role in response to biotic and abiotic stresses by activating the expression of related genes. For example, the overexpression of *StPti5*, a novel eggplant *ERF* transcription factor gene, could improve resistance to *Verticillium dahliae* and activate genes linked to the ethylene signaling pathway in *Arabidopsis thaliana* [15]. The ERF transcript factors regulated the expression of genes by directly binding to the promoters of their target genes or forming protein complexes with other transcript factors [6,16]. *OsDREB2B*, a member of the ERF family transcription factors, regulates the expression of *OsAP2-39* and interacted with *OsWRKY21* to regulate GA synthesis, resulting in a negative effect on rice growth and development [17]. The ERF transcription factor *OsRPH1* interacts with the blue light receptor Cryptochrome 1 (*OsCRY1b*), resulting in a significant reduction in plant height [18]. Additionally, ERF transcription factors can synergistically interact with hormone-signaling pathways to form a cross-regulatory network, participating in growth and development as well as abiotic stress responses mediated by abscisic acid (ABA), gibberellin (GA), auxin (IAA), ethylene (ET), brassinosteroids (BR), and cytokinin (CTK) [19,20,21,22]. The overexpression of *OsERF71* enhanced drought tolerance by regulating the expression of ABA response and proline synthesis genes, thereby reducing water loss [23]. The *OsERF096* regulates the response to low temperature in rice by modulating the accumulation and signal transduction of IAA [24]. However, not all ERF transcription factors induce biotic and abiotic stress tolerance in plants. The overexpression of certain *ERFs* in plants made them more susceptible to stress [25,26]. Members of the ERF transcription factor family act as connecting factors in the cross-regulatory network in response to stress signals [27]. Numerous studies have shown that diverse stimuli can elicit the expression of ERF transcription factors. For instance, maize *ERF* genes are induced by salt, drought, and waterlogging to mount a defense response against environmental stress [28,29,30,31]. It has been shown that the members of the DREB subfamily can recognize drought response elements and cold response elements, thereby enhancing plant resistance to abiotic stresses [8].

Nitrogen (N) is an essential element for plants, functioning in plant growth and development processes [32,33]. When confronted with high N levels, N starvation, or changes in N forms, numerous ERF family members were overexpressed in response to N perturbation [34,35,36]. In addition, many investigations have indicated that ERF transcription factors are involved in the absorption and assimilation of N [37,38]. PvAP2-1, a member of the ERF family in *P. vulgaris*, has been suggested as an essential regulator in the legume–rhizobia nitrogen-fixing symbiosis [39]. Within the ERF family, the transcription factor MdDREB2A interacts with DRE cis-elements of the MdNIR1 promoter, positively regulating nitrogen utilization [37]. Meanwhile, MdDREB2A can also directly bind to the MdSWEET12 promoter, promoting nitrogen assimilation [37]. The ERF transcription factor ZmEREB97 directly targets and regulates the expression of six *ZmNRT* genes, enhancing absorption of nitrate and nitrogen utilization efficiency [38]. Overall, the ERF family is an important transcription factor family involved in regulating nitrogen uptake and assimilation.

In plants, numerous ERF transcription factors have been identified in various species, including *Arabidopsis thaliana* [7], *Zea mays* [40], *Morus notabilis* [41], and *Osmanthus fragrans* [42]. For example, 125 *ERFs* have been identified in *Dimocarpus longan* [43], and 49 *ERFs* have been identified in *Taxus media* [44]. The poplar is widely distributed around the world and plays a significant role in various sectors, including bioenergy, timber production, and carbon sequestration, owing to its rapid growth characteristics [45,46]. Although the ERF family members have been identified in poplar species, including *Populus simonii* × *P. nigra* [13], *P. trichocarpa* [47], and *P. alba* × *P. glandulosa* [9], the tissue-specific expression patterns and the responses to nitrogen and seasonal changes of ERF family members in *P. trichocarpa* remain unclear. With the upgrade of sequencing technology, the genome of *P. trichocarpa*, a model tree species, has been sequenced multiple times, and the genomic databases are constantly being updated [48]. Therefore, it is crucial to investigate the tissue-specific expression patterns and the responses to nitrogen and seasonal changes of ERF transcription factors in *P. trichocarpa*. In this study, we systematically identified the members of the ERF family in *P. trichocarpa* and analyzed the developmental evolution relationships and gene duplication events. In addition, we analyzed the expression patterns of ERF family members in various tissues and seasons, as well as the responses to nitrogen. Therefore, this study provides a theoretical basis for exploring the regulation mechanisms of plant growth and development and responses to nitrogen by *PtERFs*.

## 2. Materials and Methods

### 2.1. Identification of the ERF Transcription Factors in P. trichocarpa

The genome sequence and protein sequence data of *P. trichocarpa* were acquired from JGI Phytozome database v13 (https://phytozome-next.jgi.doe.gov/info/Ptrichocarpa_v4_1, accessed on 10 December 2024), and the *Arabidopsis thaliana* ERF full-length protein sequences were obtained from The *Arabidopsis* Information Resource (TAIR; https://www.arabidopsis.org/, accessed on 10 December 2024). The identification of *PtERFs* in *P. trichocarpa* was performed according to the previously reported methods [49,50]. First, the amino acid sequences of the AtERF transcription factor family were retrieved from TAIR. Subsequently, a local BLASTP was conducted to obtain the homologous proteins in *P. trichocarpa* according to the AtERF amino sequences, and all sequence information with an E-value less than 10^−5^ was retained. Then, the hidden Markov model (HMM) containing the AP2 domain was used to search from the *P. trichocarpa* protein database, retaining all sequence information with an E-value less than 10^−5^. After deduplicating the amino acid sequences obtained from BLASTP and HMMER (http://www.hmmer.org/, accessed on 10 December 2024), the candidate sequences were submitted to NCBI and HMMER for verification of the AP2 domain. Finally, 210 PtERF sequences were retained.

### 2.2. Chromosomal Distribution and Bioinformatics Analysis

Using the PhytoMine tool on the Phytozome (https://jgi.doe.gov, accessed on 11 December 2024) website, amino acid sequence analysis was performed by inputting 210 ERF protein IDs to obtain the number of amino acids, loci, and chromosomal location of the ERF transcription factor family. Molecular weight, theoretical isoelectric point (pI), and grand average of hydropathicity (GRAVY) were predicted using ProtParam (https://web.expasy.org/protparam/, accessed on 11 December 2024). The chromosomal distribution of *PtERFs* was mapped using TBtools (v 2.056) [51]. Subcellular localization analysis of the ERF transcription factors was conducted using WOLF-PSORT (https://www.genscript.com/wolf-psort.html, accessed on 11 December 2024).

### 2.3. Phylogenetic and Sequence Analysis

The 210 PtERF sequences were aligned using ClustalX (version 2.1), and a phylogenetic tree of the PtERF gene family proteins was constructed using the maximum likelihood method in MEGA software (version 11.0.13) [52]. The phylogenetic tree was visualized on the iTOL website (https://itol.embl.de, accessed on 12 December 2024) [53]. The gene and protein structures of PtERFs were visualized using TBtools (v 2.056) [54,55]

### 2.4. Analysis of Gene Replication Events

MCScanX software (version 11.0.13) was employed to identify gene pairs within the *P. trichocarpa* genome and between the *A. thaliana* genome based on protein sequences. The Ka/Ks values of gene duplication pairs were calculated using TBtools. The collinearity maps of the PtERF gene family members in *P. trichocarpa* and *A. thaliana* were generated by the Circos tool in TBtools (v 2.056) [54,55].

### 2.5. Analysis of Cis-Acting Elements

Regions 2000 bp upstream of the coding sequences of PtERF members were selected using the Gtf/Gff3 Sequences Extractor in TBtools software (v 2.056). Subsequently, the cis-acting elements were analyzed and predicted using the PlantCARE database (https://bioinformatics.psb.ugent.be/webtools/plantcare/html/, accessed on 20 December 2024) [56].

### 2.6. Gene Expression Analysis

The gene expression data for the *PtERF* gene family in various tissues under different treatments, as well as in root tips and stems during different seasons, were retrieved from the Phytozome database (https://data.jgi.doe.gov/refine-download/phytozome?q=Populus+trichocarpa, accessed on 11 December 2024) under filenames of Ptrichocarpa_533_v4.1.gene_atlas_v2.meanExpression.FPKM.csv.gz and Ptrichocarpa_533_v4.1.gene_atlas_v2.meanExpression.TPM.csv.gz [57]. The heatmaps were generated using RStudio (version 2023.12.1).

### 2.7. Co-Expression Network Analysis

The co-expression genes of each *PtERF* were obtained from the Phytozome website. Genes that exhibited a correlation coefficient greater than 0.95 and had a connection count exceeding 100 were selected for constructing the PtERFs’ co-expression network. The co-expression network was then visualized using Cytoscape software (version 3.8.2). The analyses of Gene Ontology (GO) and the Kyoto Encyclopedia of Genes and Genomes (KEGG) were conducted with R (version 2023.12.1) as suggested previously [58].

## 3. Results

### 3.1. Identification and Physicochemical Properties of the PtERF Gene Family Members

**Figure 1:**
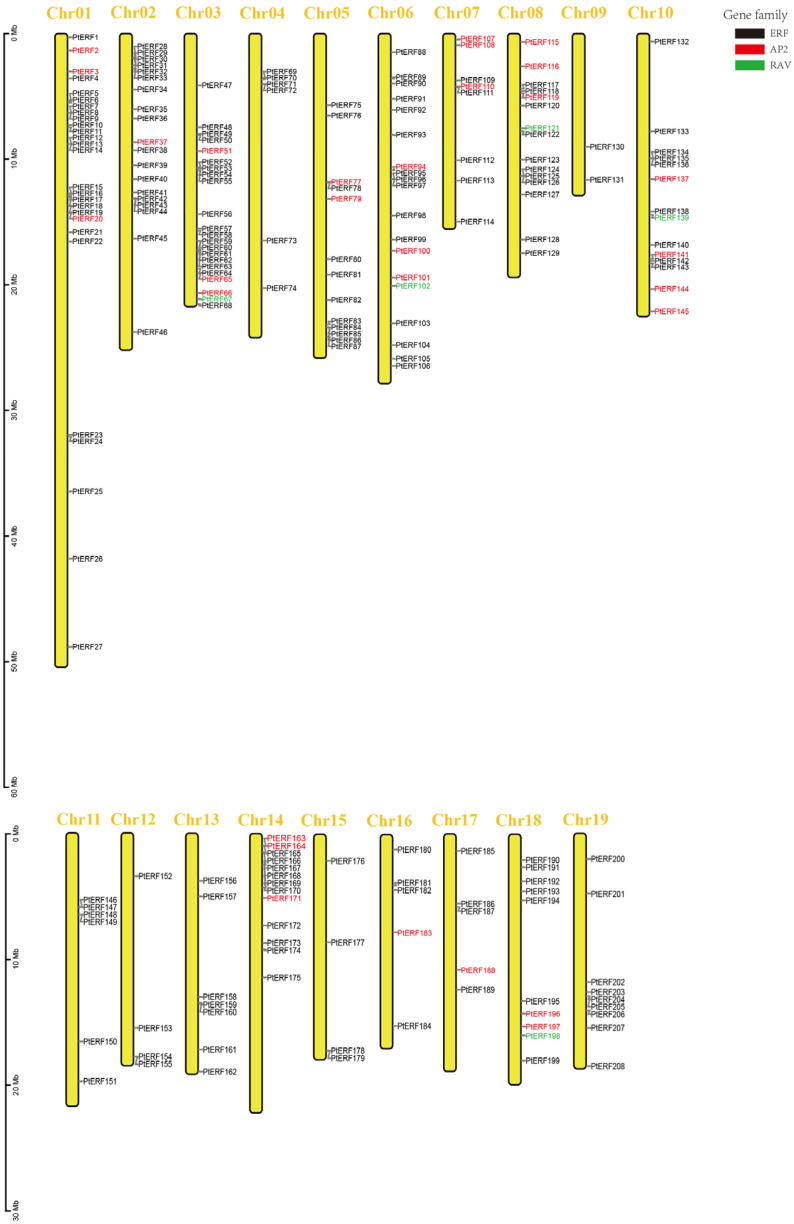
Distribution of the *ERF* genes in *Populus trichocarpa*. Different colored genes represent different subfamilies. Black: ERF subfamily; red: AP2 subfamily; green: RAV subfamily.

Based on the results of BLASTP and HMMER, a total of 210 PtERF proteins were identified in the *P. trichocarpa* (V4.1) genome database, including 176 ERF subfamily members, 29 AP2 subfamily members, and 5 RAV subfamily members (Table S1). Among the 210 *PtERFs*, 208 *PtERFs* were unevenly distributed across all 19 chromosomes while two *PtERFs* were distributed on large segments in the *P. trichocarpa* genome (Figure 1). The 210 *PtERFs* were predominantly distributed on Chr01 (12.8%), Chr03 (10.5%), Chr06 (9.0%), and Chr08 (7.1%), with the least distribution on Chr09 (1.0%). The ERF subfamily members are distributed across all chromosomes, while AP2 subfamily members are found on all chromosomes except for Chr04, Chr09, Chr11, Chr12, Chr13, Chr15, and Chr19. RAV subfamily genes have a more scattered distribution, with five members located on Chr03, Chr06, Chr08, Chr10, and Chr18 (Figure 1).

The protein sequences of 210 PtERF gene family members underwent physicochemical analysis, revealing significant variations in terms of amino acid count, molecular weight, pI, and GRAVY (Table S1). The amino acid numbers of the 210 PtERF gene family members ranged from 111 aa (PtERF70) to 721 aa (PtERF119) (Table S1). It is noteworthy that the members of the AP2 subfamily exhibit a significant increase in amino acid length compared to those of the other two subfamilies. The molecular weight of the 210 PtERF gene family members ranged from 11.99 kDa (PtERF70) to 79.23 kDa (PtERF119) (Table S1). Among the 210 members of the PtERF family, 36.19% of the proteins exhibited pIs greater than 7, while 63.81% of proteins had values less than 7. These data indicate that the majority of PtERF family proteins are acidic proteins. Moreover, the GRAVY values for 210 PtERF family proteins were below 0, indicating that they are all hydrophilic proteins. Subcellular localization analyses of PtERFs revealed that 190 PtERF proteins were found in the nucleus, 16 in the chloroplast, 3 in the cytosol, and 1 in the mitochondrion (Table S1).

### 3.2. Phylogenetic and Structural Analysis of the PtERF Gene Family Members

**Figure 2:**
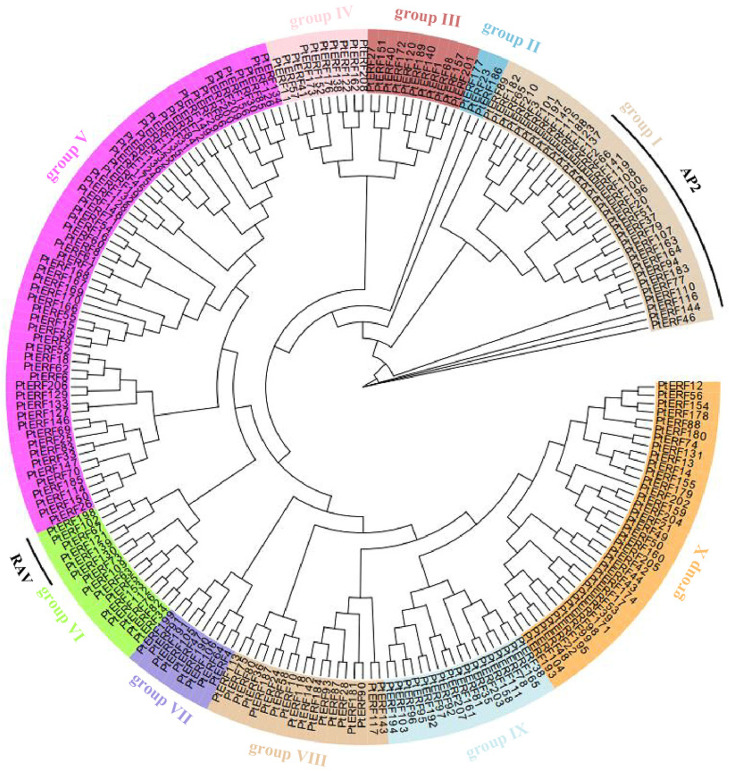
Phylogenetic analysis of *ERFs* in *P. trichocarpa*. Different colored sections represent different groups.

A phylogenetic tree was constructed using 210 PtERF protein sequences to analyze their phylogenetic relationships (Figure 2). The 210 members of the *PtERF* gene family were categorized into 10 groups, labeled groups I to X, according to their phylogenetic relationships. Members of the *ERF* subfamily were distributed across these 10 groups, while all members of the AP2 subfamily were in group I, and members of RAV subfamily were in group VI (Figure 2). Group II contained the fewest proteins, and group V was the largest subgroup (59 members), followed by group X (35 members) and group VIII (33 members) (Figure 2). Groups IX and X were closely clustered together among these 10 groups, indicating a strong phylogenetic correlation between the two groups.

By analyzing the protein and gene sequences of PtERFs, the protein and gene structures of each member are depicted in Figure S1. Upon gene structure analysis, it was found that 64 genes in the *PtERF* family contain introns, with 36 genes (17%) comprising two or more introns (Figure S1). The members of the AP2 subfamily contained six or more introns and exons, which were characterized by relatively concise 5′ and 3′ untranslated regions (UTR) (Figure S1). All members of RAV subfamily, except for *PtERF102*, lack introns. Additionally, *PtERF198* exhibits the longest 5′-UTR, while other members have either a short or absent 5′-UTR. Most members of the *ERF* subfamily contain only one exon without any introns. The analysis of protein structures demonstrates that the members of the AP2 subfamily contain two AP2 domains, members of the ERF subfamily contain only one AP2 domain, and the RAV subfamily members comprise one AP2 and one B3 domain (Figure S1).

### 3.3. Duplication Events Analysis of PtERF Gene Family

**Figure 3:**
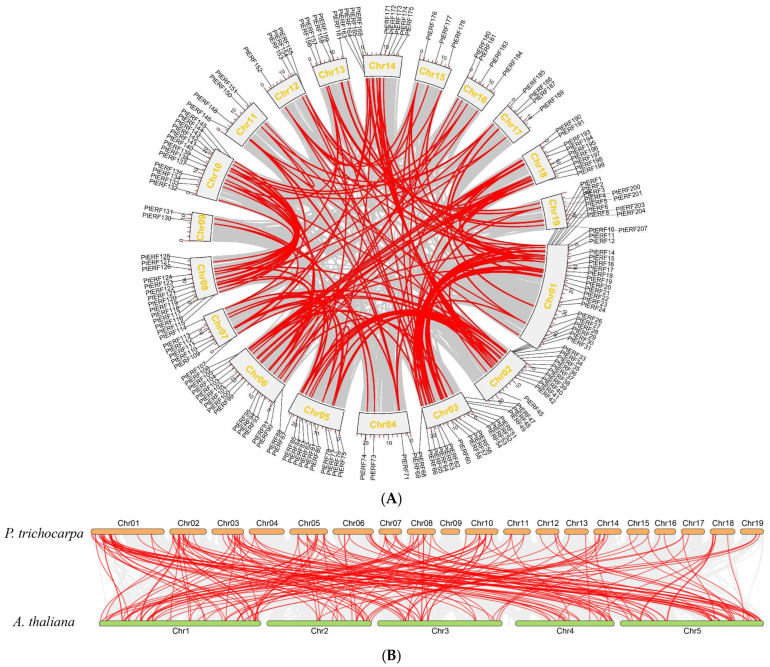
Collinearity analysis of *ERFs* in *P. trichocarpa* (**A**) and among different plant species (**B**). (**A**) Collinearity relationships of *PtERFs* in *P. trichocarpa*. (**B**) Collinearity relationships of *ERFs* between *P. trichocarpa* and *A. thaliana*. The gray lines indicate the collinearity in the genomes of *P. trichocarpa* and among different plant species. Red lines highlight the collinearity of *ERFs*.

To further explore the relationships among *PtERF* genes, we conducted gene pair calculation and collinearity analysis on *PtERF* family members (Figure 3A). A total of 168 duplicate pairs were identified among 161 *PtERF* genes, and all identified duplicates belonged to segmental duplications (Table S2). Each of *PtERF11*, *PtERF22*, *PtERF29*, *PtERF41*, *PtERF57*, *PtERF69*, *PtERF85*, *PtERF126*, *PtERF134*, *PtERF146*, *PtERF152*, *PtERF156*, *PtERF173*, *PtERF176*, *PtERF185*, *PtERF195*, and *PtERF200* were involved in five segmental duplications (Table S2). In addition, repetitive events exclusively occurred within the same subfamily, with no instances of repetition detected across different subfamilies (Figure 3A). According to the calculation of 168 *PtERF* gene duplications, the Ka/Ks values ranged from 0.02 to 0.51 (Table S2), indicating that most *PtERFs* had undergone significant purifying selection in their evolutionary history. Furthermore, between 118 *PtERF* genes and 101 *AtERF* genes, a total of 200 duplicate pairs were detected (Figure 3B).

### 3.4. Cis-Acting Element Analysis of PtERF Gene Family

**Figure 4:**
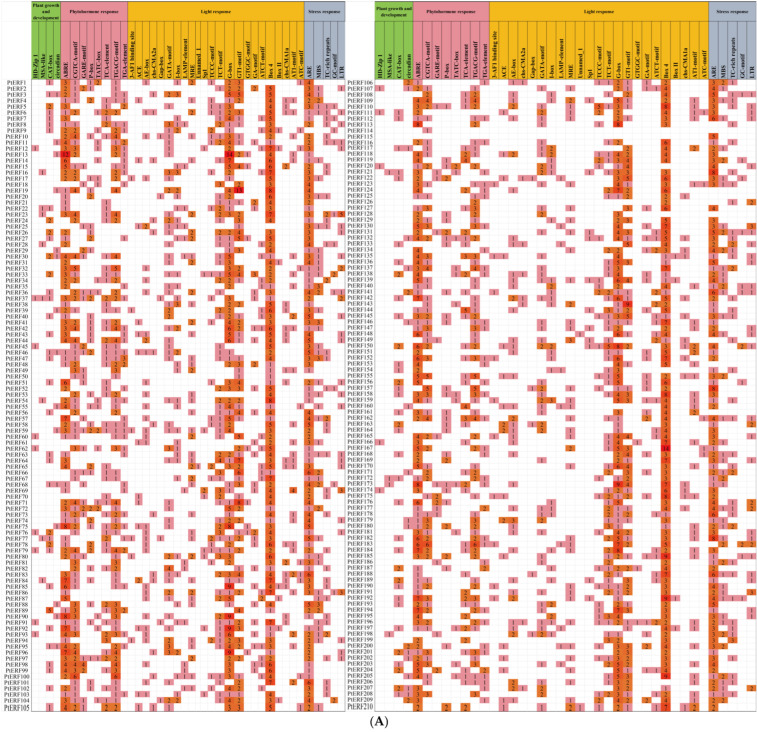

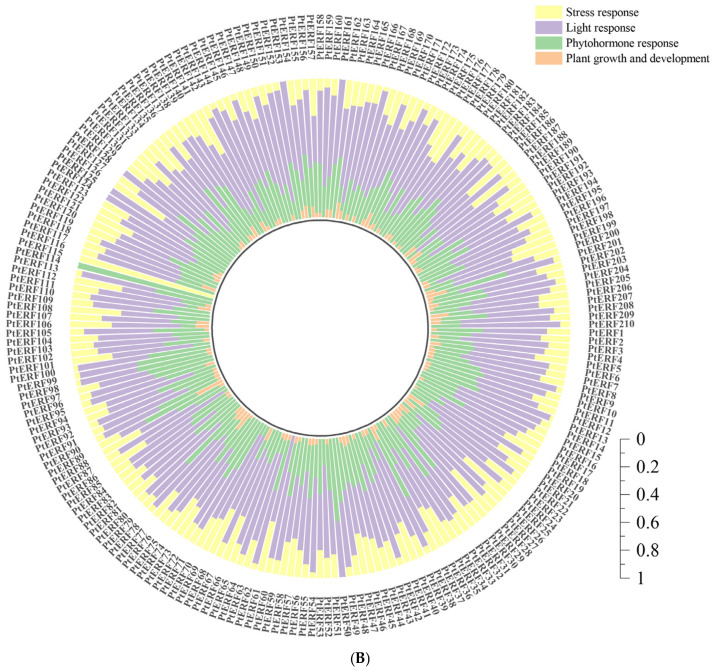
The cis-acting elements analysis of *PtERFs* in *P. trichocarpa*. (**A**) The categories and quantities of the cis-acting elements in *PtERFs*. (**B**) The percentages of the four types of the cis-acting elements in *PtERFs*. The number of different promoter elements in the *PtERFs* is represented by different intensity colors and numbers. The different colors in the histogram represent the percentage of cis-acting elements within each of the four functional categories.

To investigate the potential regulation of *PtERF* genes, the cis-acting elements of *PtERF* promoters were analyzed (Figure 4, Table S3). The results showed that many cis-acting elements were associated with light response, phytohormone response, stress response, and plant growth and development (Figure 4A). As for plant growth and development elements, most *PtERFs* contain CAT-box and circadian motifs, which are involved in the meristem expression and circadian control, respectively (Figure 4A). For phytohormone response elements, most *PtERFs* contain ABRE, which is involved in abscisic acid response; CGTCA-motif and TGACG-motif, which are involved in the MeJA response; TGA-element, which is involved in auxin response; P-box, which is involved in gibberellin response; and TCA-element, which is involved in salicylic acid response. These results indicate that *PtERFs* might be regulated by multiple hormone-signaling pathways (Figure 4A). For light-response elements, most *PtERFs* contain GATA-motif, TCT-motif, G-box, GT1-motif, and Box 4, all of which are involved in light responsiveness, indicating that *PtERFs* may be regulated by light signaling (Figure 4A). For stress response elements, most *PtERFs* contain ARE motifs involved in anaerobic induction, MBS motifs involved in drought-inducibility, TC-rich repeats involved in defense and stress responsiveness, and LTR motifs involved in low-temperature responses, implying that *PtERFs* may be involved in multiple stress responses (Figure 4A). Among the detected cis-acting elements, light-responsive cis-acting elements constituted the largest proportion, ranging from approximately 20.83% to 81.82% of all cis-acting elements identified in *PtERFs* (Figure 4B). Additionally, the promoters of *PtERF150*, *PtERF19*, and *PtERF183* contained 50, 45, and 43 cis-acting elements, respectively, suggest potential plentiful roles of these three genes in physiological processes. Taken together, the obtained data suggest that *PtERFs* play an important role in light response, phytohormone response, stress response, and plant growth and development.

### 3.5. Tissue-Specific Expression Analysis of PtERF Genes in P. trichocarpa

**Figure 5:**
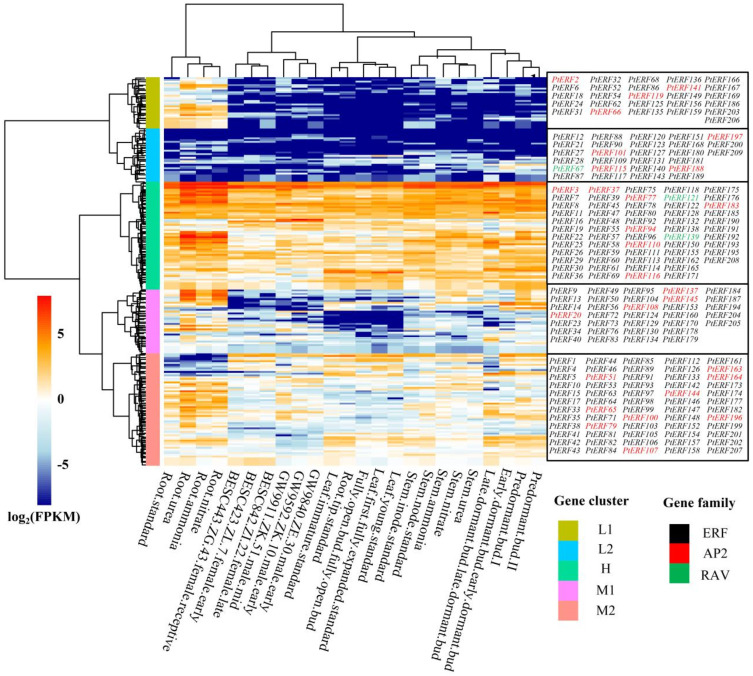
The expression of *PtERFs* in different tissues and under different nitrogen treatments. Based on the expression patterns, *PtERF* genes were classified into low-expression clusters (L1 and L2), medium-expression clusters (M1 and M2), and a high-expression cluster (H). Red bars indicate upregulation, and blue bars indicate downregulation. Different colored labels represent different subfamilies. Black: ERF subfamily; red: AP2 subfamily; green: RAV subfamily. BESC443, BESC423, BESC842, GW9840, GW9592, and GW9911 represent distinct varieties of *P. trichocarpa*.

The expression levels of the *PtERF* genes in different tissues, seasons, and treatments were derived from the Phytozome database (Figure 5). Based on the expression patterns of *PtERF* genes in different tissues under various N treatments, 210 *PtERF* genes were classified into low-expression clusters (L1 and L2), medium-expression clusters (M1 and M2), and a high-expression cluster (H) (Figure 5). The expression levels of *PtERF* genes in the roots were significantly higher compared to other tissues, while they exhibited relatively lower levels in young leaves and root tips, as observed in clusters L1, H, and M1 (Figure 5). Furthermore, among the various nitrogen treatments, the expression abundances of the *PtERF* genes in roots were found to be highest in the nitrate and urea treatment groups, while they exhibited the lowest levels in the control group (Figure 5). However, the expression of *PtERF* genes in stems was not altered among different nitrogen treatments (Figure 5). These results suggest that *PtERF* genes may be implicated in the nitrogen responses in poplar roots.

**Figure 6:**
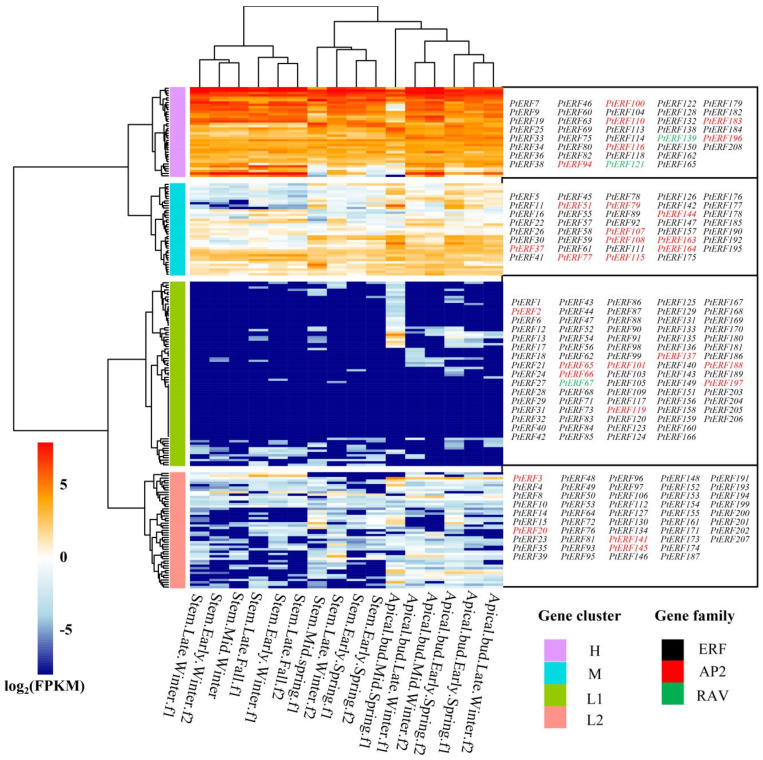
The expression of *PtERFs* in different tissues and under different seasons. Based on the expression patterns, *PtERF* genes were classified into low-expression clusters (L1 and L2), a medium-expression cluster (M), and a high-expression cluster (H). Red bars indicate upregulation, and blue bars indicate downregulation. Different colored labels represent different subfamilies. Black: ERF subfamily; red: AP2 subfamily; green: RAV subfamily.

Similarly, the expression patterns of 210 *PtERF* genes in stems and apical buds were analyzed in different seasons. These genes were then divided into low-expression clusters (L1 and L2), a medium-expression cluster (M), and a high-expression cluster (H) based on their expression levels (Figure 6). The expression levels of *PtERF* genes in stems exhibited higher magnitudes compared to those in apical buds within the same season. The expression levels of the *PtERF* genes in apical buds were highest in mid-winter f2, gradually decreasing as the seasons transitioned to late winter f1, late winter f2, early spring f1, and early spring f2 before reaching the lowest point in mid-spring f1 (Figure 6). Interestingly, the expression of the *PtERF* genes in stems also exhibited seasonal variations. The expression profiles of *PtERF* in stems gradually increased during late autumn f1, early winter f1 and f2, and mid-winter and reached a peak in late winter f1. Subsequently, the expression levels declined gradually during late winter f2 and early spring f1 and f2, reaching their lowest levels in mid-spring f1 (Figure 6). These results indicate that the expression of *PtERF* genes is influenced by seasons, with the highest expression level observed in winter. It is speculated that low temperatures may induce the expression of the *PtERFs* genes.

### 3.6. Co-Expression Analysis of PtERF Genes

**Figure 7:**
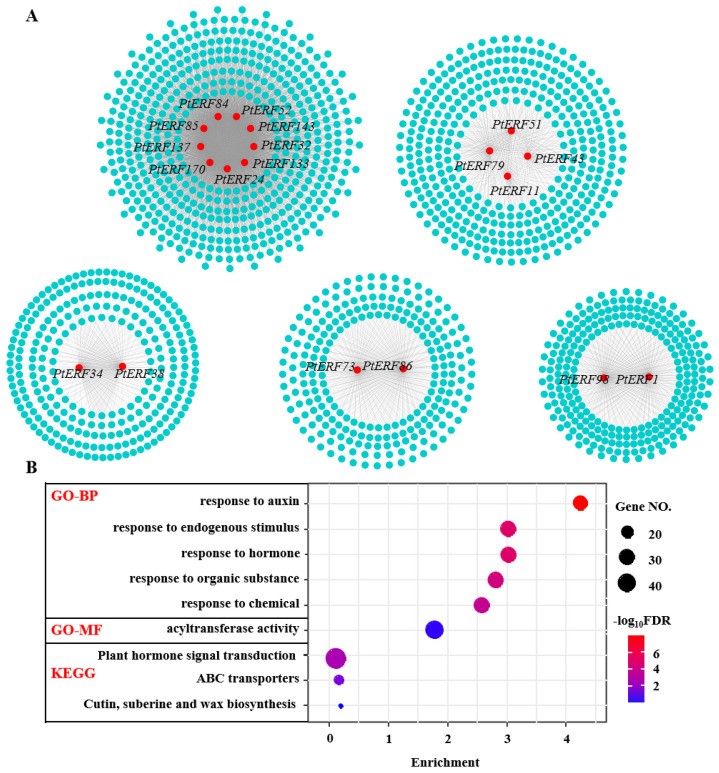
Co-expression network of *PtERFs* in *P. trichocarpa* (**A**), as well as the GO and KEGG enrichment analyses of genes within the co-expression network (**B**). In the co-expression network, red nodes represent *PtERFs*, and light blue nodes represent their co-expressed genes. The edges of the network indicate the co-expression relationships between *PtERFs* and their co-expressed genes. GO-BP and GO-MF, respectively, refer to biological process and molecular function in GO analysis.

A transcriptional co-expression network was constructed to uncover the potential biological functions played by *PtERFs*, which consisted of 1662 genes with 6440 interactions (Figure 7A). The co-expression network contained 19 *PtERF* genes and was clustered into five modules (Figure 7A). Among the 19 *PtERF* genes, *PtERF51* and *PtERF137* were classified as members of the AP2 subfamily, while the other 17 genes were categorized within the ERF subfamily. A total of 9 *PtERFs* (*PtERF52*, *PtERF143*, *PtERF32*, *PtERF133*, *PtERF24*, *PtERF170*, *PtERF137*, *PtERF85*, and *PtERF84*) and 514 co-expression genes formed the largest module (Figure 7A). Four *PtERFs* (*PtERF51*, *PtERF79*, *PtERF11*, and *PtERF43*) together with 384 co-expression genes formed the middle module. The three modules, consisting of *PtERF34* and *PtERF38*, *PtERF98* and *PtERF1*, and *PtERF73* and *PtERF86*, respectively, encompassed a total of 262, 255, and 234 co-expressed genes. Interestingly, none of shared genes were observed within the five modules. The GO and KEGG enrichment analysis of genes in the *PtERFs* co-expression network shown in Figure 7B implied roles of the *PtERFs* in plant hormone signal transduction, acyltransferase activity, response to chemicals, response to organic substances, response to hormones, response to endogenous stimuli, and response to auxin.

## 4. Discussion

The ERF family, which is a prominent transcription factor family in plants, plays a pivotal role in responding to abiotic stress, regulating the synthesis of secondary metabolites, and plant growth and development. In this study, a total of 210 *PtERF* family members were identified in the *P. trichocarpa* genome, which is larger than that in other plant species such as *Morus notabilis* (106 *ERF* genes) [41], *Arabidopsis* (147 *ERF* genes) [6], *Citrus junos* (119 *ERF* genes) [59], *Boehmeria nivea* (138 *ERF* genes) [11], and *Solanum melongena* (178 *ERF* genes) [60]. The numerous members of the *PtERF* family may be attributed to gene duplication within the subfamily, although *ERF* family members have been identified in some poplar species, such as *P. alba* × *P. glandulosa* (209 *ERF* genes) [9] and *P. trichocarpa* (209 *ERF* genes) [13]. However, as sequencing technology advances, *P. trichocarpa*, as the first poplar species to be sequenced, has undergone repeated re-sequencing and refinement of its genome data, which might result in variations in the number of *ERF* family members within *P. trichocarpa*. For example, only 200 *ERF* genes were identified in *P. trichocarpa* according to the genome of black cottonwood version 1.1 [47]. In this study, 210 *ERF* genes were identified in *P. trichocarpa* based on the genome V4.1.

Physico-chemical analysis indicated that all PtERF proteins were hydrophilic, and the majority of them were acidic proteins (Table S1). The *PtERF* genes exhibited non-uniform distribution across the 19 chromosomes, with the highest prevalence observed on Chr01. The analyses of protein and gene structures revealed significant variations among the AP2, ERF, and RAV subfamilies in terms of amino acid length, as well as the number of exons and introns. The AP2 subfamily exhibited longer gene and amino acid sequences, with a noticeable increase in both exons and introns compared to the ERF and RAV subfamilies. Notably, the majority of *ERF* family members exhibited an absence of introns, which is consistent with previous research. Among the 122 *ERF* genes in *Arabidopsis* and the 155 *ERF* genes in potato, only 20 and 25 of the *ERF* genes possess introns, respectively [6]. The 5′-UTR and 3′-UTR of the AP2 subfamily exhibited relatively short lengths, while in the RAV subfamily, apart from *PtERF198*, which possessed a longer 5′-UTR, the remaining members displayed either extremely short or absent 5′-UTRs. Genes exhibiting similar gene and protein structures were predominantly observed within the same subfamily and phylogenetic group, thereby supporting the classification of *PtERFs*. Analysis of gene duplication events suggested that segmental duplications may have contributed to the expansion of the *ERF* gene family in *P. trichocarpa*, and *PtERF* genes may have originated from three different ancestors as duplication events occurring only within subfamilies. The Ka/Ks values of duplication pairs indicated that the *PtERFs* had undergone purifying selection in evolutionary history. Based on previous study, the functional divergence of *PtERF* genes predates the emergence of dicotyledonous and monocotyledonous plants [6]. In the course of evolution, purifying selection occurs when genes attain an optimized state to ensure stability in both structural domain sequences and functions, thereby upholding the consistency and constancy of gene function throughout the evolutionary process. Therefore, the *PtERF* genes may have reached the optimized state during evolution, suggesting a potential decrease in the number of family members in future evolutionary processes.

The association of ERF transcription factors with plant growth regulation, response to low temperature, and response to nutritional stress have been extensively demonstrated by numerous studies [24,34,38,61,62,63]. For instance, *AtERF12* inhibited the accumulation of auxin, thereby modulating both root growth and leaf senescence [64]. In this study, most *PtERF* promoters contain ABRE, CGTCA-motif, TGACG-motif, TGA-element, P-box, and TCA-element, which are associated with phytohormone responsiveness; ARE, MBS, TC-rich repeats, and LTR are cis-acting elements associated with stress responses. These results showed that *PtERF* genes may be regulated to play a critical role in stress response. Additionally, the GO and KEGG analysis of co-expressed genes of *PtERFs* revealed a significant enrichment of genes involved in plant hormone signal transduction, response to auxin, response to hormones, and response to endogenous stimuli, suggesting that *PtERFs* potentially regulate plant growth through intricate hormonal signaling networks. The overexpression of *ClRAP2.4*, a member of the ERF transcription factor family, enhanced tolerance to low temperatures in Chrysanthemum lavandulifolium [61]. The season-specific expression of *PtERF* genes showed that the abundance of these *PtERF* genes was associated with seasons, with the highest levels in winter and the lowest levels in mid-spring. Hence, the induction of *PtERF* genes was speculated to be triggered by low temperatures, suggesting their potential involvement in response to cold stress [65,66]. Numerous *ERF* transcription factors were induced by nitrogen in rice and regulated leaf senescence [63]. Combined with the high expression of *PtERF* genes in roots treated with nitrate and urea, it was evident that *PtERF* genes may function in response to nitrogen. The tissue-specific expression analysis of *PtERFs* showed that the levels of *PtERF* genes were highly expressed in roots and varied significantly among different tissues, which was consistent with previous studies in longan [43] and tartary buckwheat [67].

Salt stress is one of the crucial environmental stresses that has a negative impact on plant growth and development [68]. In *Arabidopsis*, the *aterf98* mutant exhibited sensitivity to salt stress, whereas the *AtERF98*-overexpressing plants demonstrated salt tolerance by activating ascorbic acid synthesis [69]. Additionally, some *ERF* genes have been demonstrated to augment salt stress tolerance, for example, *ERF1-V* in wheat [70], *SlERF84* in tomato [71], *AtERF34* in *Arabidopsis* [72], and *ERF38* in 84K poplar [73]. The co-expression network demonstrated that *PtERF98* and *PtERF1*, as well as *PtERF34* and *PtERF38*, regulated the expression of 262 and 255 related genes, respectively. These findings suggest that *PtERF34*, *PtERF38*, *PtERF98*, and *PtERF1* may enhance plant salt tolerance by regulating the expression of these co-expressed genes.

## 5. Conclusions

A total of 210 *PtERF* genes were identified in the *P. trichocarpa* genome, which exhibited an uneven distribution across all 19 chromosomes, with the majority (90.5%) located in the nucleus. A total of 168 gene duplication events were identified in the *PtERF* gene family, all of which were categorized as fragment duplications. The Ka/Ks values for all duplicated gene pairs were less than 1, indicating a prevalence of purifying selection during evolution. Numerous cis-acting elements were identified, which were involved in responses to light, phytohormones, and stress, as well as in plant growth and development. Transcriptional analysis revealed diverse gene expression profiles of the *PtERF* gene family in response to disparate environmental stresses in different tissues. The expression levels of the *PtERF* genes were highest in roots compared to other tissues. Additionally, the expression of *PtERF* genes can be induced by nitrate, urea, and low temperature. The co-expression network centered on *PtERF* genes revealed that these *PtERFs* play crucial roles in multiple biological processes, including plant hormone signal transduction, acyltransferase activity, responses to chemical and organic substances, hormonal responses, responses to endogenous stimuli, and auxin signaling. These results lay a foundation for investigating the roles of *PtERFs* in response to nitrogen and environmental stress.

## Data Availability

Data are contained within the article and Supplementary Materials.

## Supplementary Materials

The following supporting information can be downloaded at: https://www.mdpi.com/article/10.3390/life15040550/s1, Figure S1. The analysis of gene structure and conserved domain of ERFs in *P. trichocarpa*; Table S1. Physicochemical properties and subcellular localizations of the PtERF gene family; Table S2. The Ka/Ks ratio of duplicated *PtERF* genes; Table S3. The cis-acting element analysis of *PtERFs* in 2 kb promoter region.
