# Supplementary material for: Genome-Wide Identification and Expression of the ERF Gene Family in Populus trichocarpa and Their Responses to Nitrogen and Abiotic Stresses
Source: Life (Basel). 2025 Mar 27;15(4):550. doi: 10.3390/life15040550 (PMC12029025; doi:10.3390/life15040550)
Supplement: Supplementary file 1 [file life-15-00550-s001.zip › Supplementary Figure V1.pdf]

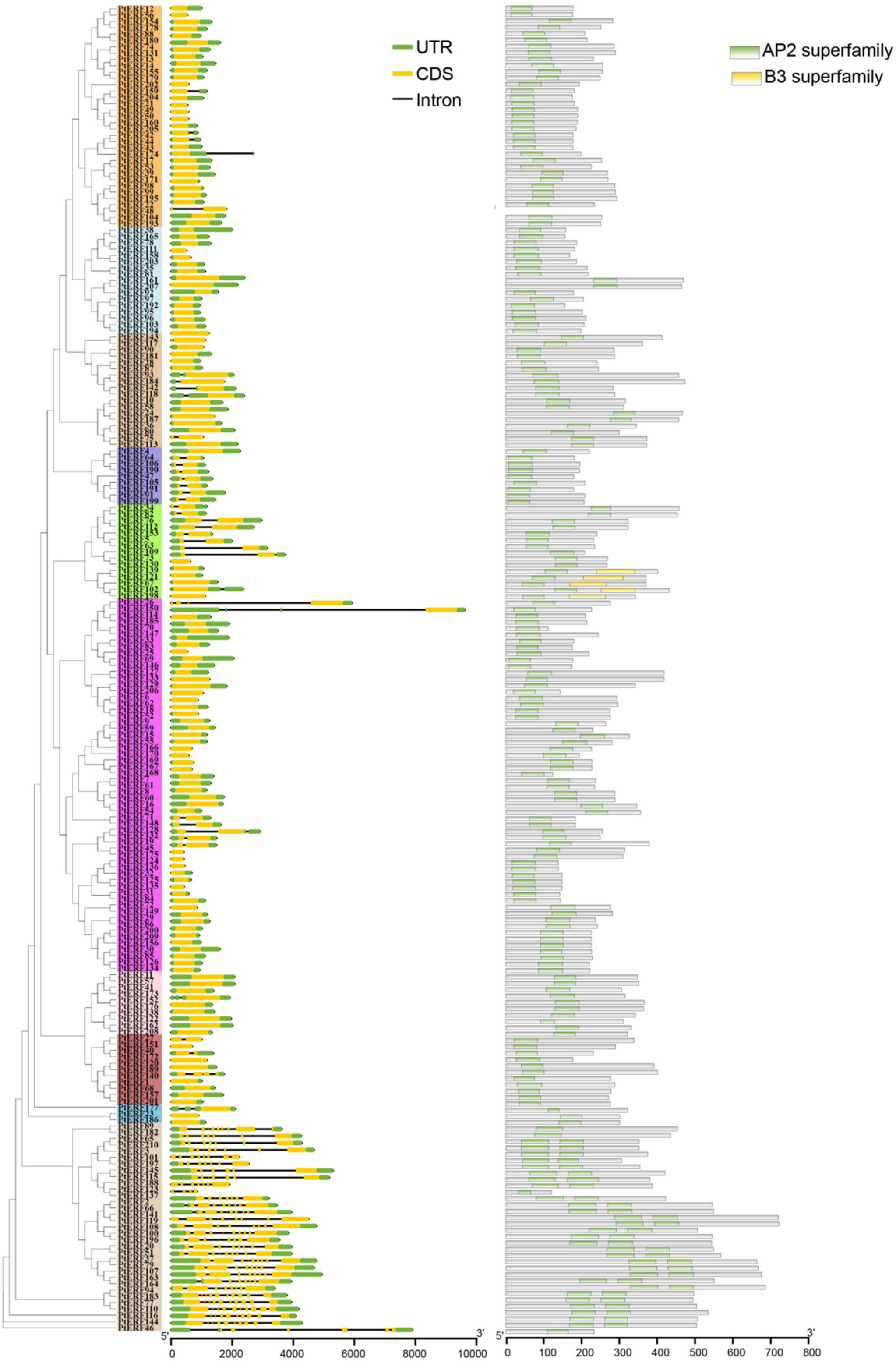

Figure S1. The analysis of gene structure and conserved domain of ERFs in *P. trichocarpa*. Different colored sections represent different groups.
